# PAK6 Phosphorylates 14-3-3γ to Regulate Steady State Phosphorylation of LRRK2

**DOI:** 10.3389/fnmol.2017.00417

**Published:** 2017-12-14

**Authors:** Laura Civiero, Susanna Cogo, Anneleen Kiekens, Claudia Morganti, Isabella Tessari, Evy Lobbestael, Veerle Baekelandt, Jean-Marc Taymans, Marie-Christine Chartier-Harlin, Cinzia Franchin, Giorgio Arrigoni, Patrick A. Lewis, Giovanni Piccoli, Luigi Bubacco, Mark R. Cookson, Paolo Pinton, Elisa Greggio

**Affiliations:** ^1^Department of Biology, University of Padova, Padova, Italy; ^2^School of Pharmacy, University of Reading, Reading, United Kingdom; ^3^Department of Morphology, Surgery and Experimental Medicine, University of Ferrara, Ferrara, Italy; ^4^Laboratory for Neurobiology and Gene Therapy, KU Leuven, Leuven, Belgium; ^5^Université de Lille, Institut National de la Santé et de la Recherche Médicale, CHU Lille, UMR-S1172, JPArc, Centre de Recherche Jean-Pierre AUBERT Neurosciences et Cancer, Lille, France; ^6^Institut National de la Santé et de la Recherche Médicale, UMR-S 1172, Team “Early Stages of Parkinson's Disease”, Lille, France; ^7^Department of Biomedical Sciences, University of Padova, Padova, Italy; ^8^Proteomics Center, University of Padova and Azienda Ospedaliera di Padova, Padova, Italy; ^9^Center for Integrative Biology, University of Trento, Trento, Italy; ^10^Laboratory of Neurogenetics, National Institute on Aging/NIH, Bethesda, MD, United States

**Keywords:** PAK6, 14-3-3, LRRK2, Parkinson's disease, phosphorylation

## Abstract

Mutations in Leucine-rich repeat kinase 2 (LRRK2) are associated with Parkinson's disease (PD) and, as such, LRRK2 is considered a promising therapeutic target for age-related neurodegeneration. Although the cellular functions of LRRK2 in health and disease are incompletely understood, robust evidence indicates that PD-associated mutations alter LRRK2 kinase and GTPase activities with consequent deregulation of the downstream signaling pathways. We have previously demonstrated that one LRRK2 binding partner is P21 (RAC1) Activated Kinase 6 (PAK6). Here, we interrogate the PAK6 interactome and find that PAK6 binds a subset of 14-3-3 proteins in a kinase dependent manner. Furthermore, PAK6 efficiently phosphorylates 14-3-3γ at Ser59 and this phosphorylation serves as a switch to dissociate the chaperone from client proteins including LRRK2, a well-established 14-3-3 binding partner. We found that 14-3-3γ phosphorylated by PAK6 is no longer competent to bind LRRK2 at phospho-Ser935, causing LRRK2 dephosphorylation. To address whether these interactions are relevant in a neuronal context, we demonstrate that a constitutively active form of PAK6 rescues the G2019S LRRK2-associated neurite shortening through phosphorylation of 14-3-3γ. Our results identify PAK6 as the kinase for 14-3-3γ and reveal a novel regulatory mechanism of 14-3-3/LRRK2 complex in the brain.

## Introduction

P21 activated kinase 6 (PAK6) is a member of the group II PAK serine/threonine kinases, highly conserved enzymes containing a kinase domain at the carboxyl-terminus and a Cdc42 and Rac-interactive binding (CRIB) domain at the amino-terminus (Kumar et al., 2017). PAKs play a central role in signal transduction and one of the best-characterized functions of these kinases is their role in actin cytoskeleton reorganization *via* the LIM kinase-cofilin pathway (Kumar et al., 2017). In contrast to class I PAKs (PAK1-3) which are activated by Rho GTPase (e.g., Rac1) binding, class II PAKs (PAK4-6) are relocalized by Rho GTPases at specific signaling sites and locally activated by binding with specific partners containing SH3 domains (Ha et al., 2012). Given the importance of cytoskeletal organization in neuronal development and function, accumulating studies investigated the role of PAKs in the nervous system and their implication in neurodegenerative diseases (Civiero and Greggio, 2017). *Pak6* knock-out mice are viable and fertile with no overt neurological defects, whereas the double *Pak5/Pak6* knockout mice exhibit cognitive and locomotor activity deficits, and cultured neurons display reduced neurite length and number (Nekrasova et al., 2008). Although PAK6 is almost exclusively expressed in the brain, the molecular details of PAK6-mediated pathways in neuronal cells are largely unexplored.

14-3-3s constitute a family of adaptor proteins participating in multiple processes within in the cell. Dimeric 14-3-3s bind to their partners at specific phospho-sites (mainly phospho-serines) and assist protein localization, activity and stability (Sluchanko and Gusev, 2017). 14-3-3s are themselves phosphorylated at multiple sites the main ones being Ser58/59, Ser184, and Ser/Thr232 (https://www.phosphosite.org; Aitken, 2011), although the upstream kinases are poorly characterized. The breakpoint cluster region protein (BCR) interacts with at least five isoforms of 14-3-3 *in vivo* and phosphorylates 14-3-3τ on Ser233 and to a lesser extent 14-3-3ζ on Thr233 (Clokie et al., 2005). Instead, protein kinase A (PKA) phosphorylates 14-3-3ζ at Ser58 and PKA-mediated phosphorylation leads to modulation of 14-3-3ζ dimerization affecting its interaction with partner proteins (Gu et al., 2006). Functionally, 14-3-3s intervene in the regulation of small G-proteins and protein kinases related to the proper functioning of the cytoskeleton (Sluchanko and Gusev, 2010). Of note, they have been nominated as group II PAK interactors and Ser99 phosphorylation within PAK4 generates a binding site for 14-3-3γ that promotes the formation of a PAK4-LIMK1 complex during cell migration (Bastea et al., 2013).

Recently, we described a novel role for PAK6 in mammalian brain. We found that PAK6 kinase activity promotes neurite outgrowth through interaction with the Parkinson's disease (PD)-associated leucine-rich repeat kinase 2 (LRRK2) (Civiero et al., 2015). LRRK2 belongs to the family of ROCO proteins, characterized by the presence of the bidomain ROC (Ras of complex proteins) and COR (c-terminal of ROC; Civiero et al., 2014). ROC functions as a GTPase and represents a signaling output, whereas COR operates as a dimerization device (Guaitoli et al., 2016). Another signaling output in LRRK2 is a serine-threonine kinase domain, which undergoes both autophosphorylation and heterologous phosphorylation (Greggio et al., 2009; Sheng et al., 2012). The majority of PD-associated mutations increase kinase activity in cells and *in vivo* (Sheng et al., 2012; Steger et al., 2016), resulting in neuronal toxicity and, in the case of the common G2019S mutation, decreased neurite length and complexity (MacLeod et al., 2006; Sepulveda et al., 2013; Matikainen-Ankney et al., 2016). LRRK2 undergoes autophosphorylation *in vitro* and *in vivo* (Greggio et al., 2008; Sheng et al., 2012), and it is phosphorylated by other kinases, including casein kinase 1 alpha (CK1α), IκB kinases (IKKs), and PKA, within a cluster of N-terminal serine residues (Ser910, Ser935, Ser955, Ser973) and at S1444 in the ROC domain (Dzamko et al., 2012; Chia et al., 2014; Muda et al., 2014). Toll-like receptor agonists increase LRRK2 phosphorylation at Ser935 (Dzamko et al., 2012), whilst LRRK2 inhibitors abolish auto- and heterologous phosphorylation causing LRRK2 relocalization into discrete intracellular clusters (Dzamko et al., 2010). Phosphorylation of Ser910, Ser935, Ser955, and Ser973 in the N-terminus and Ser1444 in the ROC domain promote 14-3-3 binding to LRRK2, which protects LRRK2 from protein phosphatase 1 (PP1)-dependent dephosphorylation and consequent LRRK2 relocalization (Dzamko et al., 2010; Lobbestael et al., 2013; Muda et al., 2014). While these residues are not autophosphorylation sites, their phosphorylation status depend on LRRK2 kinase inhibitors, suggesting that LRRK2 may control a kinase(s) or phosphatase(s) regulating its phosphorylation cycle. Thus, the cellular phosphorylation of LRRK2 seems implicated in its subcellular localization and activation through binding with 14-3-3 proteins through a yet unknown regulatory mechanism.

By investigating the PAK6 interactome, we found that PAK6 binds and phosphorylates 14-3-3γ at Ser59 *in vitro* and in cells. Phosphorylation at this specific site induces 14-3-3γ monomerization, resulting in decreased affinity for client proteins. Furthermore, we demonstrate that PAK6-dependent phosphorylation of 14-3-3γ, an established LRRK2 interactor, promotes 14-3-3γ/LRRK2 complex dissociation *in vitro* and in cells with consequent LRRK2 dephosphorylation at Ser935. In neurons, PAK6 activation rescues the neurite shortening induced by the pathogenic G2019S LRRK2 form in a process that is dependent on phospho-Ser59 of 14-3-3γ. Collectively, our work uncovers PAK6 as the kinase of 14-3-3γ and discloses a novel pathway by which this enzyme promotes neurite development in neuronal cells.

## Materials and methods

### Animals

C57BL/6 LRRK2 wild-type and (mouse) LRRK2 G2019S BAC mice were obtained from Jackson Laboratory [B6.Cg-Tg(Lrrk2^*^G2019S)2Yue/J]. Housing and handling of mice were done incompliance with national guidelines. All animal procedures were approved by the Ethical Committee of the University of Padova and the Italian Ministry of Health (license 1041/2016-PR).

### Plasmids

Eukaryotic expression contructs of 3xFlag tagged PAK6 (wild type, K436M and S531N) and 3xFlag LRRK2 (wild type and G2019S) were generated as described previously (Civiero et al., 2015). All seven 14-3-3s isoforms were cloned in pET28a(+) vector in fusion with an N-terminal His-tag for expression in *E. coli* cells by PCR amplification of the sequences from mammalian expression plasmids kindly provided by Prof. Dario Alessi (University of Dundee). PAK6 and 14-3-3 mutant variants were generated using the Quick-Change II site-directed mutagenesis kit (Stratagene). For bioluminescence resonance energy transfer (BRET) analysis, pRL-TK plasmid (Promega) and pEYFP-C/N1 (Novagene) were modified with Gateway™ Vector Conversion System (Invitrogen). LRRK2 and 14–3–3γ coding sequences, respectively, were subsequently cloned inside these vectors using LR recombination reaction kit (Invitrogen). pmCherry-N1 vector (Novagen) was used to transfect neurons and trace neurites.

### Antibodies

For immunoprecipitation the following antibodies were used: mouse Flag M2 (Sigma, Cat# F1804, 1 μg/mg total proteins). For immunoblotting analysis the following antibodies were used: Flag M2, HRP conjugated (Sigma, Cat# F1804, 1:10,000), rabbit pan-14-3-3 Santa Cruz, Cat# sc-629, 1:1,000), mouse monoclonal Anti-polyHistidine, HRP conjugated (Sigma, Cat#A7058, 1:10,000), rabbit phospho-PAK4-5-6 (Sigma, Cat# SAB4504052, 1:2,000), mouse β-tubulin (Sigma, Cat# T8328, 1:5,000), rabbit pan phospho-Ser58 14-3-3 (Abcam, Cat# ab30554, 1:1,000), rabbit 14–3–3γ (ThermoFisher, Cat# PA5-29690, 1:10,000), rabbit MJFF2 (Epitomics Cat# 3514-1, 1:100), rabbit phospho-Ser935 (Epitomics Cat# 5099-1, 1:100), mouse GFP (Roche, Cat# 1181460001, 1:1,000). For immunocytochemistry analysis the following antibodies were used: rabbit PAK6 (Prestige® Sigma, Cat# HPA031124, 1:200), rabbit 14–3–3γ (ThermoFisher, Cat# PA5-29690, 1:200), rabbit pan phospho-Ser58 14-3-3 (Abcam, Cat# ab30554, 1:200).

### Chemicals

The PAK inhibitor, PF-3758309 (Selleckchem), was used for endogenous PAK inhibition at 10 μM for 90 min. PhosTag molecule was produced by MRC PPU Reagents and Services, University of Dundee. All solvents and chemicals used for LC-MS/MS analysis were of MS grade (Sigma).

### Cell cultures and transfection

HEK293T cells were cultured in Dulbecco's modified Eagle's medium supplemented with 10% fetal bovine serum. HEK293T were transfected with plasmid DNA using polyethylenimine (Polysciences) according to the manufacturer's recommendations.

### Neuronal cultures and transfection

Cortical neuron cultures were prepared from C57BL/*6* and G2019S LRRK2 BAC mouse pups (P0). Neuronal cultures were plated and grown on 12 mm diameter coverslips (400,000 cells/well) in 12-well plastic tissue culture plates as previously described (Belluzzi et al., 2016). Neuronal cultures were transfected with DNA plasmids at 3 days *in vitro* (DIV) using Lipofectamine 2000 according to the manufacturer's protocol (Thermo Fisher). Neurons were fixed using PFA 4% at DIV7 and subjected to analysis.

### Cell lysis, co-immunoprecipitation, and western blotting

Cells were harvested at 48 h post-transfection and lysed in buffer containing 50 mM Tris pH 7.5, 1% Triton X-100, 1 mM sodium orthovanadate, 5 mM sodium pyrophosphate, 50 mM sodium fluoride, 0.27 M sucrose, 1 mM ethylenediaminetetraacetic acid (EDTA). Lysates were incubated with anti-Flag M2 agarose beads (Sigma) overnight. Immunocomplexes were washed three times with lysis buffer supplemented with 0.25 M NaCl. Immunoprecipitates were resuspended in sample buffer. Between 15 and 30 μg of protein samples were resolved on pre-casted 4–20% Tris-glycine polyacrylamide gels (Biorad) or home-casted Tris-glycine polyacrylamide [stacking gel: 4% (w/v) acrylamide, 125 mM Tris/HCl, pH 6.8, 0.1% (w/v) SDS, 0.2% (v/v) *N,N,N*′*,N*′-tetramethylethylenediamine (TEMED) and 0.08% (w/v) ammonium persulfate (APS), separating gel: 7.5 or 13% (w/v) acrylamide, 375 mM Tris/HCl, pH 8.8, 0.1% (w/v) SDS, 0.1% (v/v) TEMED, and 0.05% (w/v) APS] in SDS/Tris-glycine running buffer. Precision Plus molecular weight markers (Biorad) were used for size estimation. Resolved proteins were transferred to polyvinylidenedifluoride (PVDF) membranes using semi-dry Biorad transfer machine (Trans-Blot Turbo Transfer System). The PVDF sheets were blocked in Tris-buffered saline plus 0.1% Triton (TBS-T) plus 5% nonfat dry milk for 1 h at 4°C and then incubated overnight at 4°C with antibodies in TBS-T plus 5% non-fat dry milk. The PVDF membranes were washed in TBS-T (3 × 10 min) at room temperature followed by incubation for 1 h at RT with horseradish peroxidase-conjugated anti-mouse IgG. Blots were then washed in TBS-T (4 × 10 min) at RT and rinsed in TBS, and immunoreactive proteins were visualized using enhanced chemiluminescence plus (ECL+, GE Healthcare, Little Chalfont, England). Densitometric analysis was carried out using Image J software.

### Pull-down assay

His-14-3-3 isoforms were expressed in BL21(DE3) bacterial cells and purified in batch by IMAC with ProBond™ resin (Invitrogen). After elution, imidazole was removed and buffer exchange in PBS with a PD10 desalting column (GE healthcare). After quantification, 3 mM dithiothreitol (DTT) was added for storage and proteins were quickly frozen and kept at −80°C. Instead, Flag-tagged proteins were expressed in and purified from HEK293T cell lines as previously described (Civiero et al., 2015). Flag-purified proteins bound to the resin were incubated for 2 h with purified His-14-3-3 isoforms (1 μM). For the following procedure see co-immunoprecipitation and western blotting section.

### Protein digestion, phosphopeptide enrichment, and LC-MS/MS analysis

Gel bands were subjected to in-gel digestion with sequencing-grade modified trypsin (Promega) as detailed in (Belluzzi et al., 2016). A similar procedure was used for the digestion of phosphorylated proteins, but in this case LysC was used as protease. Briefly, gel bands were cut in small pieces, dehydrated with acetonitrile (ACN) and dried under vacuum. Reduction of cysteines was performed with freshly prepared 10 mM DTT in 25 mM Tris-HCl pH 8.5, at 56°C and for 1 h. Alkylation was performed with 55 mM iodoacetamide (Sigma) in 25 mM Tris-HCl pH 8.5 for 45 min at room temperature and in the dark. Gel pieces were washed several times with 25 mM Tris-HCl pH 8.5 and ACN, dried under vacuum, and suspended LysC solution (Promega, 12.5 ng/mL in 25 mM Tris-HCl pH 8.5). Digestion was performed overnight at 37°C. Peptides were extracted with three changes of 50% ACN/0.1% formic acid (FA, Fluka). Samples were dried under vacuum and stored at −20°C till the phosphopeptide enrichment procedure was performed. Phosphopeptide enrichment and LC-MS/MS analysis were performed as detailed in (Belluzzi et al., 2016). Data were acquired with a LTQ-Orbitrap XL mass spectrometer (Thermo Fisher Scientific) interfaced to a nano-HPLC Ultimate 3000 (Dionex—Thermo Fisher Scientific). Raw data files were analyzed with Proteome Discoverer software (version 1.4, Thermo Fisher Scientific) connected to a Mascot Server version 2.2.4 (Matrix Science, UK) against the Uniprot Human Database (version 2015.04.01, 90411 sequences). Trypsin or LysC were set as digesting enzymes with up to one missed-cleavage for protein identification and up to three missed-cleavages for phosphopeptides identification. Carbamidomethyl cysteine was set as fixed modification and methionine oxidation as variable modification in all cases. Phosphorylation of Ser/Thr/Tyr residues was set as variable modifications when the phosphopeptide analysis was performed. Peptide and fragment tolerance were 10 ppm and 0.6 Da, respectively. The algorithm Percolator was used to calculate False Discovery Rate (FDR) and PhosphoRS algorithm (Taus et al., 2011) was used to help in the assignment of the correct phosphorylation sites.

### Overlay assays

To directly assess 14-3-3 interaction with targets, overlay assay were performed as described in Nichols et al. (2010). Briefly, immunopurified proteins or brain cell lysates were electroblotted onto nitrocellulose membranes and blocked with 5% skimmed milk for 30 min. After washing with TBS-T, membranes were incubated with purified His-14-3-3s diluted to 1 μg/ml in 5% bovine serum albumin (BSA) in TBS-T overnight at 4°C. Membranes were subsequently cut to allow incubations with the different 14–3–3γ forms. Subsequent western blotting and detection was done in parallel to allow quantitative comparison.

### *In vitro* kinase reactions

Kinase assays were carried out as previously described using 1 mM ATP (Civiero et al., 2012). Protein concentrations used are indicated in Figure legends.

### PhosTag gel

Samples were supplemented with 10 mM MnCl_2_ before loading gels. Phos-tag Tris-glycine polyacrylamide gel was carried out as described previously (Steger et al., 2016). Gels for Phos-tag Tris-glycine polyacrylamide gels consisted of a stacking gel [4% (w/v) acrylamide, 125 mM Tris/HCl, pH 6.8, 0.1% (w/v) SDS, 0.2% (v/v) TEMED, and 0.08% (w/v) APS] and a separating gel [7.5% (w/v) acrylamide, 375 mM Tris/HCl, pH 8.8, 0.1% (w/v) SDS, 75 μM Phos-tag acrylamide, 150 μM MnCl_2_, 0.1% (v/v) TEMED, and 0.05% (w/v) APS].

### Anion exchange chromatography

Samples belonging from *in vitro* kinase reactions and corresponding to 80 μg of recombinant 14-3-3s were loaded in a 6 ml Resource Q column (Amersham Biosciences) and eluted at 1 ml/min with a discontinuous NaCl gradient (0-0.35 M/6.5-18.5 ml, 0.35-0.4 M/18.5-30.5 ml, 0.4-1 M/30.5-33.5 ml) in 20 mM Tris pH 8 using an ÄKTA purifier system (GE Healthcare). Signals were recorded with an UV detector set at 280 nm. Fractions were collected during each run and the identity of protein peaks subsequently confirmed by dot blot.

### Blue native gels

Precast vertical SERVA*Gel*™ N Native gels (SERVA Electrophoresis) 4–16% were loaded and run according to manufacturer instructions for Blue Native electrophoresis. For each sample a total amount of 5 μg of protein was loaded. At the end, the gel was stained with Coomassie Brillant Blue.

### Immunocytochemistry and confocal imaging

Transiently transfected HEK293T or neuronal coverslips coated with poly-L-lysine were fixed with 4% paraformaldehyde (PFA). Cells were stained with the appropriate primary antibody and subsequently with secondary anti-mouse AlexaFluor® 633-conjugated (Life technologies, Cat# A-21126), anti-rabbit Alexa® 568-conjugated (Life technologies, Cat# A-11036) or anti-rabbit DyLight 405-conjugated AffinityPure (Jackson Immunoresearch Laboratories Inc, Cat# 111-475-003) antibodies were used at 1:200. Images were acquired using a Leica TCS SP5 confocal microscope or Leica 5000B fluoresce microscope.

For neurite branching analysis, all the measurements were performed using NeuronJ as previously described (Civiero et al., 2015). Neurites and dendritic spines were semi-automatically traced and quantified in terms of length and number.

### BRET

HEK293T cells were transfected with indicated expression vectors. After 48 h, cells were scraped and transferred into a white OptiPlate-96 (Perkin Elmer). Luminescent emission was stimulated by adding 5 μM Coelenterazine. After 10 min signals derived from RLuc and YFP were collected with the Victor3 plate reader (Perkin Elmer). BRET was calculated by the ratio YFP/RLuc, after to obtain net BRET (nBRET) the value of BRET derived from non trasfected cell was subtracted to the samples values.

### Statistical analysis

All quantitative data are expressed as mean ± SEM (standard error of the mean) and represent at least three independent sets of experiments. Significance of differences between two groups was assessed by Student *t-*test or by one-way ANOVA with Bonferroni's *post-hoc* test and two-way ANOVA with Tukey's *post-hoc* test when more than two groups were compared.

## Results

### PAK6 interacts with 14-3-3γ in its active state

**Figure 1:**
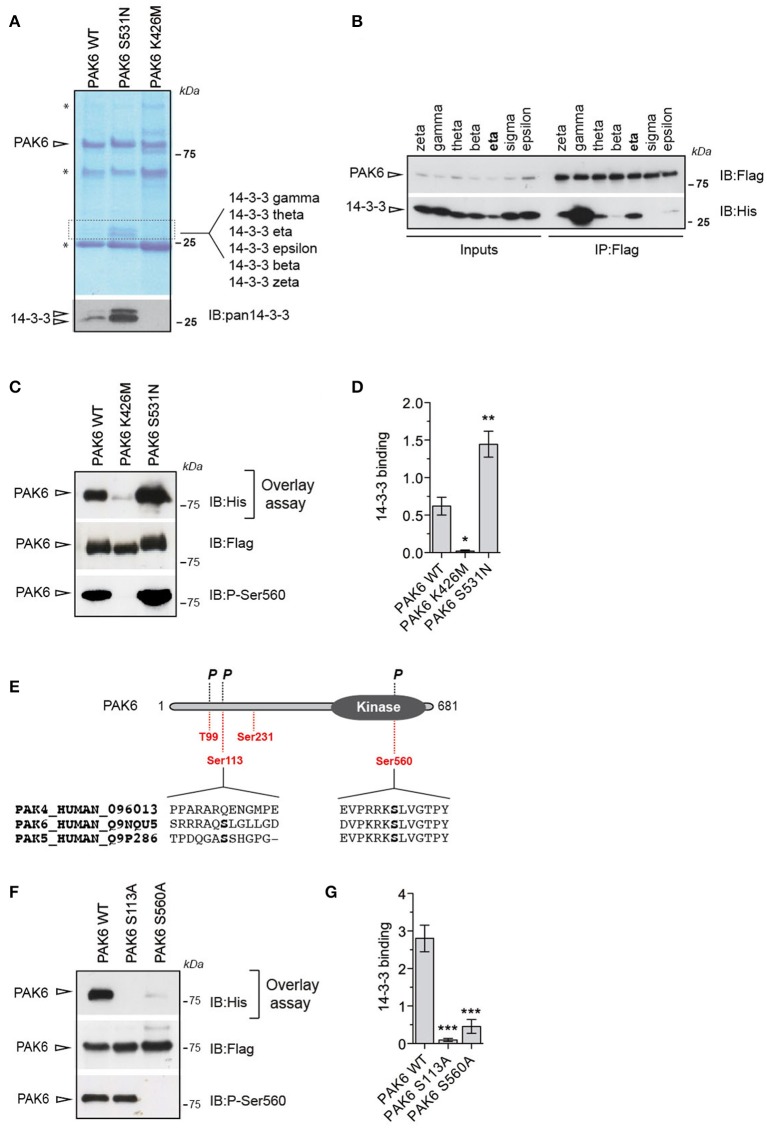
Autophosphorylated PAK6 binds 14-3-3γ **(A)** Immunoprecipitated PAK6 wild-type, S531N, and K436M were subjected to SDS-PAGE analysis and bands differentially enriched across mutants above 25 kDa were subjected to LC-MS/MS analysis. LC-MS/MS revealed that several 14-3-3 isoforms as kinase-dependent PAK6 interactors and western blot using pan-14-3-3 antibody confirmed the identity of the proteins (^*^ refers to antibody light and heavy chains). **(B)** Immunopurified Flag-PAK6 was used to performed pull-down assays with the seven recombinant 14-3-3 isoforms. Bound 14-3-3s were revealed by western blot using anti-His antibody.**(C)** Immunopurified PAK6 wild-type, K436M and S531N were loaded on a SDS-PAGE, transferred on nitrocellulose membrane and subjected to overlay assays using recombinant 14-3-3γ. Anti-Flag and anti-pSer560 antibodies were used to verify PAK6 loading and activation, respectively, and anti-His to quantify bound 14-3-3. **(D)** Quantification of bound 14-3-3γ normalized by total PAK6 amount (*n* = 3 independent experiments, one-way ANOVA followed by Bonferroni's multiple comparison test; ^*^P ≤ 0.05, ^**^P ≤ 0.01). **(E)** Schematic representation of PAK6 sequence. In red, putative 14-3-3- binding sites and phosphorylation (*P*) (https://www.phosphosite.org/). **(F)** Immunopurified PAK6 wild-type, S113A and S560A were loaded on SDS-PAGE, transferred onto nitrocellulose membranes and subjected to overlay assays using recombinant 14-3-3γ. Anti-Flag and anti-pSer560 antibodies were used to confirm PAK6 loading and activation, respectively, and anti-His to quantify bound 14-3-3. **(G)** Quantification of bound 14-3-3γ normalized by total PAK6 amount (*n* = 3 independent experiments, one-way ANOVA followed by Bonferroni's multiple comparison test; ^***^P ≤ 0.001).

To identify novel PAK6 effectors and the impact of PAK6 kinase activity on its interactome, we performed immunoprecipitation experiments in HEK293T cells using as bait 3xFlag-PAK6 wild-type and the active variant S531N. As negative control, we immunoprecipitated kinase-dead PAK6 K436M. The kinase activity of these functional mutants was previously characterized *in vitro* and in cells by our group and others (Schrantz et al., 2004; Civiero et al., 2015). After SDS-PAGE separation, coomassie staining revealed the presence of a doublet around 25 kDa that was differentially enriched across the PAK6 variants, with PAK6 S531N displaying the highest affinity and no bands visible in the PAK6 K436M immunoprecipitate (Figure 1A). The identity of the two bands was investigated by liquid chromatography/tandem mass spectrometry (LC-MS/MS) analysis, revealing multiple 14-3-3 isoforms [γ gamma, θ theta, η eta, ε epsilon, β beta, ζ zeta; Figure 1A and Supplemental File (Datasheet 2)]. Using a pan-14-3-3 antibody we confirmed that PAK6 binds 14-3-3s and that the binding increases proportionally with PAK6 kinase activity (Figure 1A). Human 14-3-3s constitute a family of seven highly conserved regulatory proteins (Berg et al., 2003). To further explore the specificity of PAK6 binding toward the different 14-3-3 isoforms, 3xFlag-PAK6 bound to anti-Flag resin was incubated with 6xHis-14-3-3s purified from *E. coli*. As shown in Figure 1B, PAK6 pulls down several 14-3-3 isoforms but displays the highest affinity for 14-3-3γ. Since 14-3-3 binding is a function of PAK6 kinase activity (Figure 1A), we then tested 14-3-3γ binding to PAK6 wild-type, S531N and K436M using overlay assays (Figure 1C). The kinase activity of PAK6 functional mutants was assessed by western blot monitoring the autophosphorylation site Ser560 (Civiero et al., 2015; Figure 1C). As expected, PAK6 S531N displays two-folds higher affinity for 14-3-3γ with respect to wild-type, whereas the inactive mutant does not bind 14-3-3 (Figures 1C,D). Dimeric 14-3-3 proteins dock onto pairs of phosphorylated serine/threonine residues on target proteins (Mackintosh, 2004). PAK6 is predicted to accommodate four potential 14-3-3 binding sites: Thr99, Ser113, Ser231, and Ser560 (https://ania-1433.lifesci.dundee.ac.uk/prediction/webserver/index.py/getting_started). Three of them (Thr99, Ser113, and Ser560) have been previously found phosphorylated in cells (http://www.phosphosite.org; Figure 1E). Among them, Thr99 is not involved in 14-3-3 binding (Tinti et al., 2014). Ser560 is a highly conserved autophosphorylation site in cells, and it is essential for PAK6 autophosphorylation activity and phosphorylation of substrates [Kaur et al., 2005; Supplemental File (Datasheet 1): Figures S1A,B]. Conversely, we found that autophosphorylation of Ser113 is not required for phosphorylation of PAK6 substrates but the ~2-fold decrease in ^33^P incorporation by PAK6 indicates that it is a *bona fide* autophosphorylation site [Supplemental File (Datasheet 1): Figures S1A,B]. Overlay assays with the phospho-deficient Ser560Ala and Ser113Ala variants indicate that both sites are required for 14-3-3 binding (Figures 1F,G), consistent with the typical 14-3-3 binding mode where the dimer docks into two distinct serine/threonine residues within its targets (Tinti et al., 2014). Collectively, these experiments indicate that 14-3-3γ is a PAK6 interactor and that the formation of 14-3-3/PAK6 complex is dependent on PAK6 autophosphorylation on Ser113 and Ser560.

### PAK6 phosphorylates a subset of 14-3-3s

**Figure 2:**
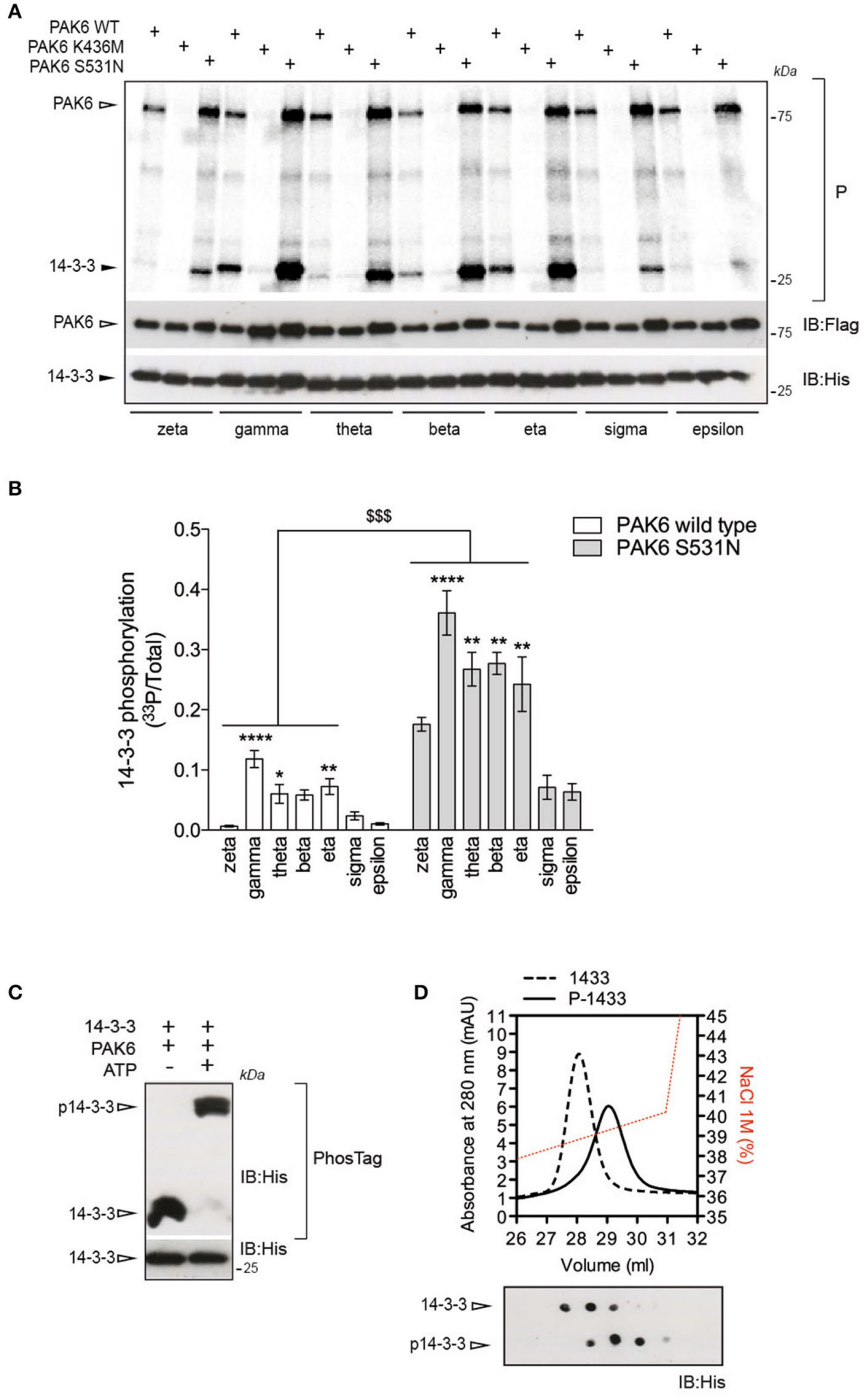
14-3-3γ is a PAK6 substrate. **(A)** Recombinant His-14-3-3 isoforms were incubated with Flag-PAK6 wild-type, K436M and S531N (4:1 molar ratio) in the presence of ^33^P-ATP/Mg^2+^ for 1 h and subjected to SDS-PAGE, transferred onto nitrocellulose membranes and imaged with a phosphoscreen. Total protein amount was verified by western blot with anti-Flag and anti-His antibodies. **(B)** Quantification of incorporated ^33^P-radioactivity normalized by total 14-3-3 (*n* = 4 experiments. Data are presented as mean ± SEM. Two-way ANOVA followed by Tukey's multiple comparison test (^*^P ≤ 0.05, ^**^P ≤ 0.01, and ^****^P ≤ 0.0001 comparison among 14-3-3 isoforms; ^$$$^*P* ≤ 0.001, comparison between PAK6 WT versus PAK6 S531N). **(C)** Purified His-14-3-3γ was incubated with Flag-PAK6 S531N (4:1 molar ratio) in the presence or absence of 1 mM ATP and subjected to PhosTag analysis to analyze phosphorylation stoichiometry and SDS-PAGE to verify total protein amount. Anti-His antibody was used to reveal 14-3-3 in both cases. **(D)** Cation exchange profiles of 14-3-3γ phosphorylation by PAK6 (back dashed line) and non-phosphorylated (black solid line) and NaCl gradient in red. On the bottom, dot blot analysis of eluted fractions using anti-His antibody.

14-3-3s are phosphorylated at multiple sites (https://www.phosphosite.org). However, little is known about the physiological significance of this phosphorylation as well as the identity of the kinases mediating this process. Interestingly, 14-3-3s contain a putative group II PAKs consensus site around Ser58/59 (II-PAKs consensus: G**G**RR**RR**R**SW**ASPGGK (Rennefahrt et al., 2007); putative consensus in 14-3-3s: ^53^V**G**A**RR**S**SW**RVISSI^66^). Therefore, to assess whether 14-3-3s are phosphorylated by PAK6, we performed radioactive kinase assays *in vitro* using recombinant 3xFlag-PAK6 wild-type, K436M and S531N and the seven 14-3-3 isoforms as substrates. PAK6 could efficiently phosphorylate a subset of 14-3-3s (including γ, η, and θ; Figures 2A,B) and PAK6 S531N increased the amount of radiolabelled 14-3-3 by three- to four-folds compared to wild-type (Figures 2A,B). Being the best PAK6 interactor and preferred substrate among the seven isoforms, we selected 14-3-3γ for subsequent investigation. First we evaluate the stoichiometry of PAK6 phosphorylation on 14-3-3γ with PhosTag gels coupled with immunoblot analysis. We found that PAK6 completely phosphorylates 14-3-3γ *in vitro* after 60 min incubation under kinase assay conditions (1 mM ATP, 1 μM PAK6, and 4 μM 14-3-3γ), as revealed by the retarded electrophoretic mobility of phosphorylated 14-3-3γ (Figure 2C). Moreover, in cation exchange chromatography, phosphorylated 14-3-3γ elutes at higher NaCl concentrations compared to the non-phosphorylated protein, consistent with an increase of the overall negative charge due to the phosphate groups (Figure 2D). Together, these findings indicate PAK6 as a kinase for 14-3-3γ *in vitro*.

### Ser59 phosphorylation of 14-3-3γ causes dimer to monomer switch and loss of affinity for the client proteins

**Figure 3:**
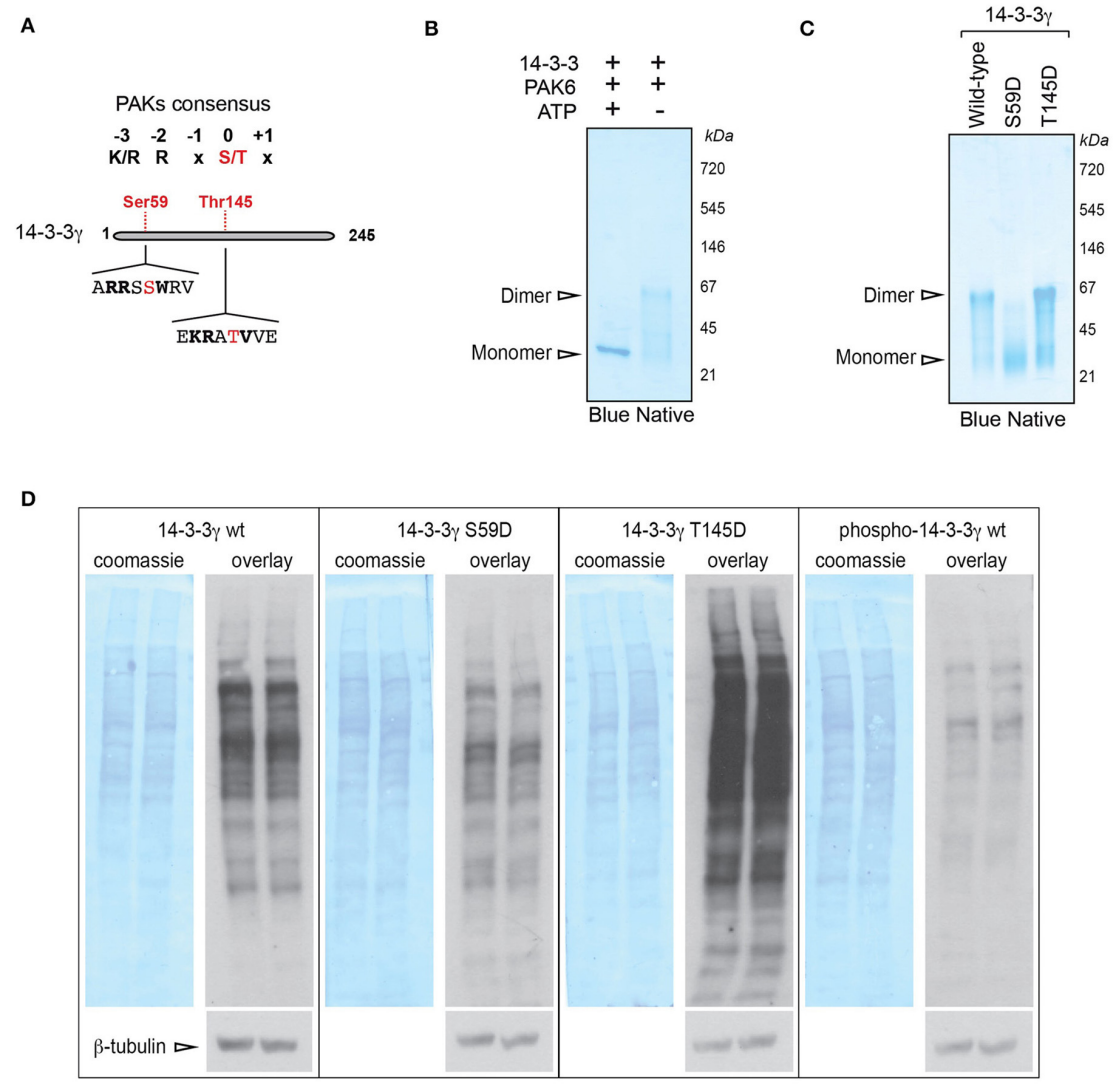
PAK6-mediated phosphorylation at Ser59 regulates 14-3-3γ affinity for target proteins. **(A)** Schematic of 14-3-3 protein sequence and selected phospho-sites identified by LC-MS/MS containing a group II PAK consensus sequence. **(B)** Recombinant His-14-3-3γ was incubated with Flag-PAK6 S531N (4:1 molar ratio) in the presence or in the absence of 1 mM ATP and subjected blue native gel separation. **(C)** Recombinant His-14-3-3 wild-type, S59D and T145D quaternary structure were investigated by blue native gel electrophoresis. **(D)** Total brain proteins were separated by SDS-PAGE, transferred onto nitrocellulose membranes and subjected to overlay assays using recombinant 14-3-3γ wild-type, S59D, T145D, and PAK6-phosphorylated (p14-3-3). Anti-His antibody, anti-βeta-tubulin and coomassie staining were used to verify bound 14-3-3 and protein loading, respectively. Representative overlay assays and western blots from *n* = 3 independent replicates. Experiments were run in two technical replicates per each condition and in parallel to allow for comparison.

To identify the site(s) phosphorylated by PAK6 on 14-3-3γ, we used phospho-peptide enrichment coupled with LC-MS/MS analysis on purified 14-3-3γ phosphorylated by PAK6 *in vitro*. Proteins were digested with LysC to obtain peptides compatible with MS/MS analysis around the predicted Ser58 phosphorylation. Under these experimental conditions, we found a number of putative phosphorylated peptides [Supplemental File (Datasheet 1): Table S1] but only two of them (^143^RA**T**VVESSEK^152^ and ^51^NVVGARR**SS**WRVISSIEQK^69^) were consistently recovered across independent MS runs [Supplemental File (Datasheet 1): Figures S2A,B]. In both peptides a group II PAK consensus sequence could be identified (Figure 3A). The peptide ^143^RATVVESSEK^152^ was predicted to be phosphorylated at the threonine residue (Thr145), whilst the peptide ^51^NVVGARRSSWRVISSIEQK^69^ was found to be phosphorylated at one of the two tandem serine residues in position 58 or 59. However, the second serine corresponding to Ser59 in 14-3-3γ is the one predicted to be recognized by class II PAKs.

Previous work suggested that phosphorylation of 14-3-3ζ at Ser58 (corresponding to Ser59 in 14-3-3γ induces dimer to monomer transition; Woodcock et al., 2003; Zhou et al., 2009). We therefore performed blue native electrophoresis to evaluate whether PAK6-mediated phosphorylation alters the dimeric state of 14-3-3γ. In the absence of ATP (but under the same kinase assay conditions), 14-3-3γ presents an electrophoretic mobility around the size of the dimer (Figure 3B). Instead, PAK6-phosphorylated 14-3-3γ separates as a sharp band around the monomer size (Figure 3B), indicating that PAK6-mediated phosphorylation on 14-3-3γ may influence its quaternary structure. The MS results also hints that Ser59 in 14-3-3γ might be a putative PAK6 phosphorylation site. To further explore the consequence of PAK6 phosphorylation on 14-3-3γ dimer/monomer, we generated 14-3-3γ phosphomimetic mutants corresponding to the two phospho-sites predicted by the MS analysis by replacing the serine residues with aspartate (S59D and T145D). To this end, 14-3-3γ wild-type, S59D and T145D were purified and loaded on blue native gels. This analysis revealed that the T145D mutant exhibits an electrophoretic motility similar to that of the wild-type protein, whereas the S59D proteins separate as a band compatible with the size of a monomer (Figure 3C). The dimer to monomer transition was proposed to decrease the affinity of 14-3-3s for their target proteins (Tzivion et al., 1998; Zhou et al., 2009). We therefore investigated the consequence of Ser58 phosphorylation on mouse brain proteome by overlay assays. Total mouse brain lysates separated by SDS-PAGE and transferred onto nitrocellulose membranes were probed with 14-3-3γ wild-type, 14-3-3γ S59D, 14-3-3γ T145D, and p14-3-3 wild-type (i.e., phosphorylated by PAK6) and processed in parallel to allow proper comparison. Overlay assays showed that 14-3-3γ wild-type and 14-3-3γ T145D bind several proteins on the membrane (Figure 3D). However, the phosphomimetic 14-3-3γ S58D and p14-3-3γ display a lower affinity for brain proteins with respect to the wild-type. Collectively, these results indicate that PAK6 phosphorylates 14-3-3γ *in vitro* in at least two sites, but only Ser59 phosphorylation causes a conformational change that decreases the affinity of 14-3-3γ for binding partners.

### PAK6 phosphorylates endogenous 14-3-3γ at Ser59

**Figure 4:**
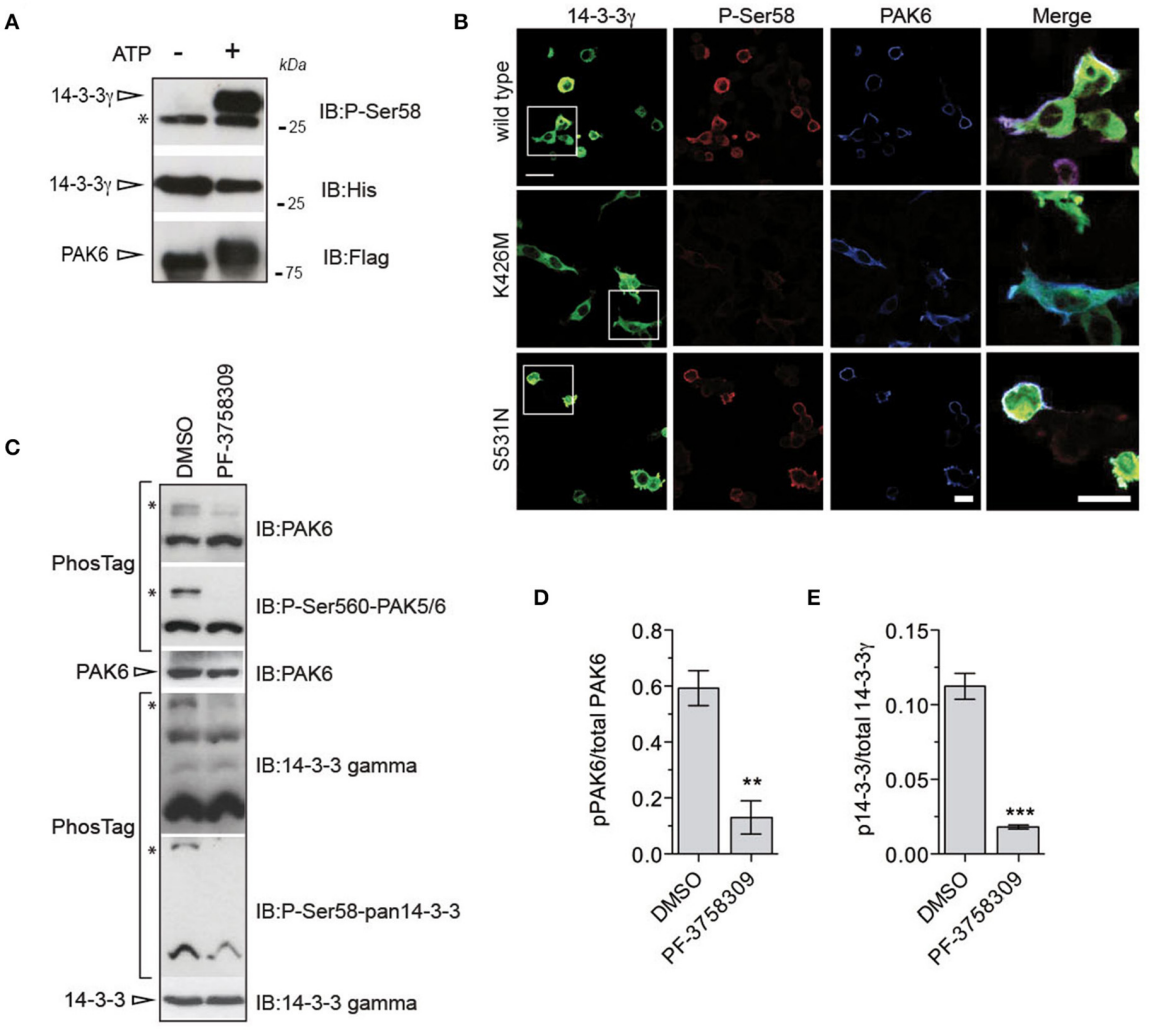
PAK6 phosphorylates 14-3-3γ at Ser59 in cells. **(A)** Recombinant His-14-3-3γ was incubated with Flag-PAK6 S531N (4:1 molar ratio) in the presence or absence of 1 mM ATP and subjected to western blot analysis. Anti-pSer59 was used to reveal the amount of phosphorylated 14-3-3 and anti-His and anti-Flag antibodies to verify 14-3-3 and total PAK6 amount. (^*^) indicates endogenous pSer58/59 revealed by the pan phospho-antibody corresponding to 14-3-3s co-precipitated with purified PAK6 (see Figure 1A). **(B)** HEK293T cells co-transfected with YFP-14-3-3γ (green) together and Flag-PAK6 wild-type, K436M, and S531N. Anti-pSer58 antibody (red) and anti-Flag (cyano) antibodies were used to visualize phosphorylated 14-3-3 and PAK6, respectively. **(C)** MEF lysates treated with 10 μM PF-3758309 and DMSO as control were loaded in a PhosTag gel. Phospho- and dephospho-proteins (PAK6 and 14-3-3γ) were visualized using anti-PAK6 or anti-pSer560 antibody and anti-14-3-3γ or anti-pSer58. Protein loading was controlled by western blot using anti-PAK6 and anti-14-3-3γ antibodies. **(D,E)** Quantification of pPAK6 normalized by total PAK6 and of p14-3-3γ normalized by total 14-3-3γ. Data are presented as mean ± SEM. ^**^P ≤ 0.01 and ^***^P ≤ 0.001 (*n* = 3 independent experiments, Student's *t*-test).

*In vitro* kinase assays and western blot analysis with pan anti-phospho Ser58 (Ser59 in 14-3-3γ) antibody confirmed that PAK6 phosphorylates 14-3-3γ at Ser59 [Figure 4A and Supplemental File (Datasheet 1): Figure S3]. To investigate whether this phosphorylation is also relevant in the cellular context, we used immunofluorescence analysis. We observed that PAK6 phosphorylates 14-3-3γ at this site in cells co-transfected with YFP-14-3-3γ and Myc-PAK6 wild-type and PAK6 S531N, with no staining in the presence of PAK6 kinase dead K436M (Figure 4B). Next, we tested whether phosphorylation also occurs on endogenous proteins. To this aim, mouse embryonic fibroblasts, which express high levels of PAK6 and 14-3-3γ, were acutely treated with the potent PAK inhibitor PF-3758309 or dimethyl sulfoxide (DMSO) and analyzed with PhosTag gels. As expected, PF-3758309 treatment efficiently inhibited PAK6 autophosphorylation as indicated by a significant reduction of the upper band that correspond to pSer560 (Figures 4C,D). Importantly, a parallel reduction of the band corresponding to 14-3-3γ phosphorylated at Ser59 was also evident in the presence of PAK6 inhibition (Figures 4C,E). Taken together, these experiments demonstrate that endogenous PAK6 phosphorylates endogenous 14-3-3γ at Ser59.

### Phospho-14-3-3γ loses affinity for LRRK2

**Figure 5:**
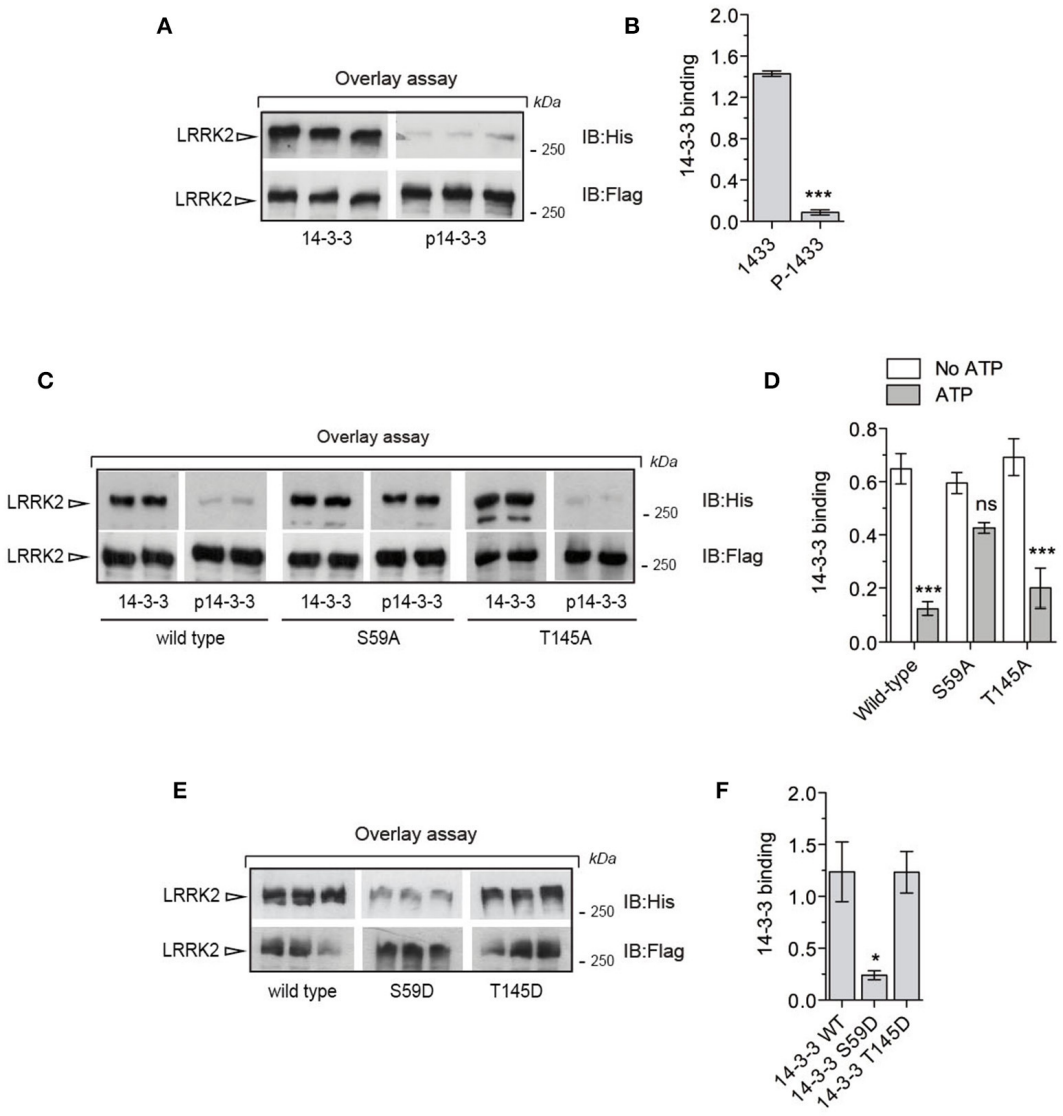
Phosphorylation of Ser59 is a switch for LRRK2-14-3-3γ dissociation. **(A)** Immunopurified Flag-LRRK2 was loaded into SDS-PAGE, transferred onto nitrocellulose membranes and subjected to overlay assays using recombinant 14-3-3γ and PAK6-phosphorylated 14-3-3γ. Anti-Flag and anti-His antibodies were used to verify LRRK2 loading and bound 14-3-3γ, respectively. SDS-PAGE/transfers were done in the same gel/membrane and membranes were subsequently cut to allow incubations with the different 14-3-3γ forms. Subsequent western blotting and detection was done in parallel to allow quantitative comparison. **(B)** Quantification of bound 14-3-3γ normalized by total LRRK2 amount (*n* = 3 independent experiments). Data are presented as mean ± SEM. ^***^P ≤ 0.001 (Student's *t*-test). **(C)** Immunopurified Flag-LRRK2 was loaded in SDS-PAGE, transferred onto nitrocellulose membranes and subjected to overlay assays using recombinant 14-3-3γ wild-type, S59A and T145A or PAK6-phosphorylated 14-3-3γ wild-type, S59A and T145A. Anti-Flag and anti-His antibodies were used to verify LRRK2 loading and bound 14-3-3γ, respectively. **(D)** Quantification of bound 14-3-3γ wild-type, S59A and T145A normalized by total LRRK2 amount. Data are presented as mean ± SEM. ^***^P ≤ 0.001 (*n* = 3 independent experiments, two-way ANOVA followed by Tukey's multiple comparison test). **(E)** Immunopurified Flag-LRRK2 was loaded in SDS-PAGE, transferred onto nitrocellulose membranes and subjected to overlay assays using recombinant 14-3-3γ wild-type, S59D and T145D. Anti-Flag and anti-His antibodies were used to verify LRRK2 loading and bound 14-3-3γ, respectively. **(F)** Quantification of bound 14-3-3γ wild-type, S59D and T145D normalized by total LRRK2 amount. Data are presented as mean ± SEM. ^*^P ≤ 0.05 (*n* = 3 independent experiments, one-way ANOVA followed by Tukey's multiple comparison test).

So far our data suggest that phosphorylation of 14-3-3γ at Ser59 regulates the affinity for its binding partners. Since 14-3-3s are well established and characterized interactors of the kinase LRRK2, we asked whether PAK6-mediated phosphorylation of 14-3-3 impacts LRRK2 binding. Using overlay assays, we first tested whether PAK6 and LRRK2 share common 14-3-3s interactors. We observed that 14-3-3 zeta, gamma and theta display high affinity for both PAK6 and LRRK2 compared to others isoforms [Supplemental File (Datasheet 1): Figure S4]. Previous studies indicate that 14-3-3γ is involved in the regulation of cytoskeletal dynamics (Bastea et al., 2013), a process also linked with LRRK2-PAK6 pathway (Civiero et al., 2015). In addition, 14-3-3γ was found to be a LRRK2 interactor by other groups (Li et al., 2011; Muda et al., 2014) and it is the preferential PAK6 substrate and interactor across the seven isoforms. Based on this reasoning, we assessed the effect of PAK6-mediated phosphorylation of 14-3-3γ on LRRK2 binding with overlay assays. First, 14-3-3γ was incubated with PAK6 S531N in the presence or absence of 1 mM ATP as previously described (Figure 2C) and subsequently applied in parallel to nitrocellulose membranes containing equal amounts of purified LRRK2. We observed a striking reduction of phospho-14-3-3γ affinity for LRRK2 compared to non-phosphorylated proteins, suggesting that phosphorylation may regulate 14-3-3:LRRK2 complex formation (Figures 5A,B). To substantiate this finding, we generated alanine mutants of the two candidate phospho-residues identified by LC-MS/MS (S59A and T145A) and subjected them to PAK6 phosphorylation in the presence of 1 mM ATP or without nucleotide as control. We observed that pre-phosphorylation of 14-3-3 reduces its interaction LRRK2 in wild-type and T145A proteins but has no effect in the S59A variant (Figures 5C,D). As a further evidence, the phosphomimetic S59D mutant exhibits ~80% reduced binding capacity toward LRRK2 compared to wild-type and T145D 14-3-3γ, phenocopying the effect of PAK6 phosphorylation (Figure 5E,F). Taken together, these experiments reveal that PAK6-dependent phosphorylation of 14-3-3γ at S59 disrupts 14-3-3:LRRK2 complex *in vitro*.

### PAK6 regulates LRRK2-14-3-3 binding in cells with consequent modulation of LRRK2 phosphorylation at Ser935

**Figure 6:**
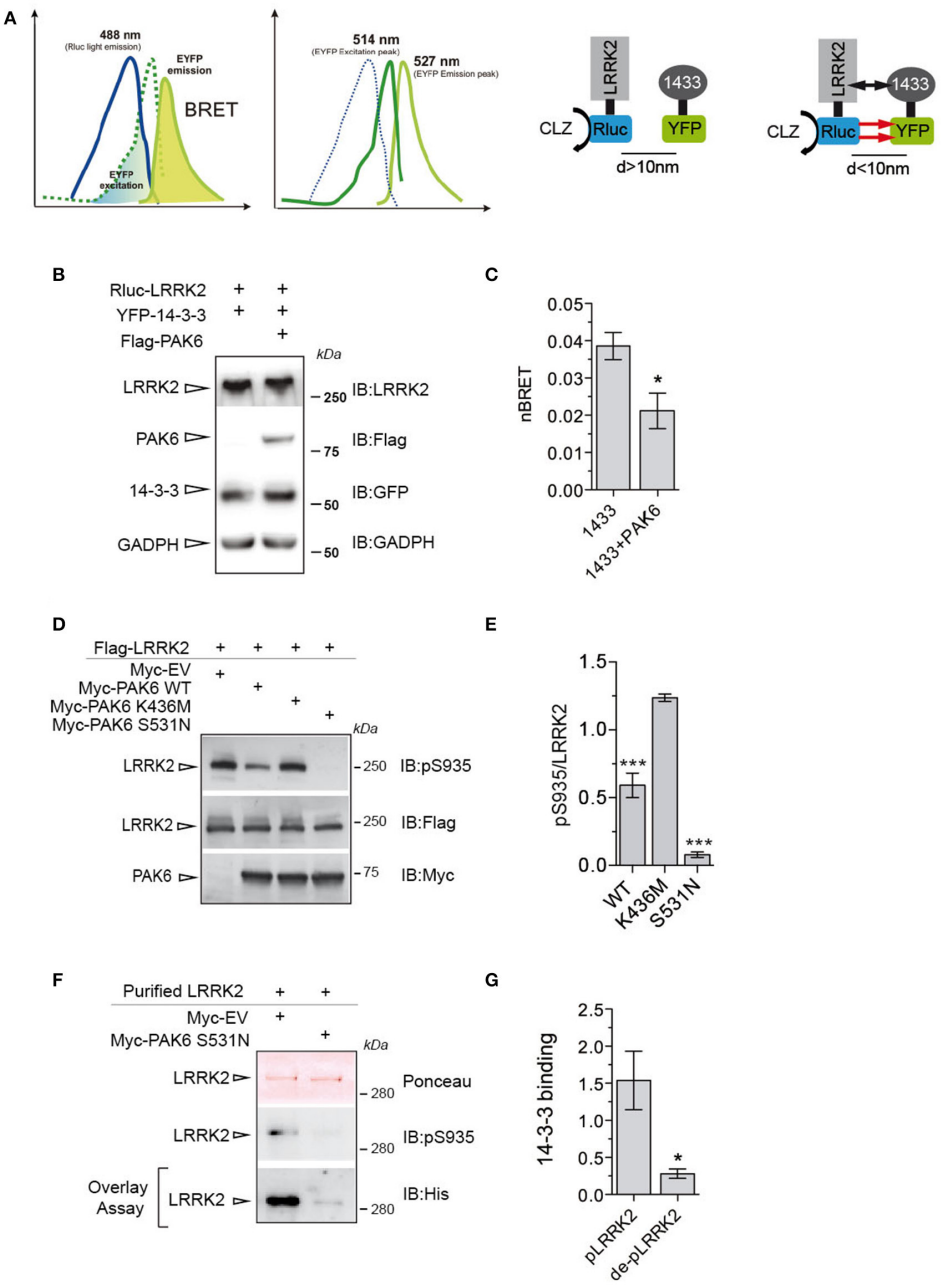
PAK6 causes LRRK2-14-3-3γ dissociation in cells resulting in LRRK2 de-phosphorylation. **(A)** BRET uses a bioluminescence luciferase (RLuc) to produce an initial photon emission (488 nm) compatible with the fluorecence acceptor (YFP) (514 nm), upon the addition of RLuc substrates (coelenterazine). The following constructs were cloned: LRRK2-RLuc (donor) and YFP-14-3-3γ (acceptor). BRET signal was calculated as described in the Materials and Methods section. **(B)** Cells were transfected with LRRK2-RLuc, YFP-14-3-3γ and Flag-PAK6. Anti-LRRK2, anti- Flag, anti-GFP and anti-GADPH were used to verify protein amount. **(C)** nBRET signals calculated between LRRK2 and 14-3-3γ in the absence and in the presence of Flag-PAK6 overexpression (*n* = 4 independent experiments, Student's *t*-test analysis; ^*^P ≤ 0.05). **(D)** HEK293T cells were transfected with Flag-LRRK2 together with Myc-empty vector (EV), Myc-PAK6 wild-type, K436M and S531N and lysates loaded in SDS-PAGE and pSer935-LRRK2 analyzed by western blot using anti-Ser935 antibody and anti-Flag and anti-Myc antibodies to verify LRRK2 and PAK6 amount. **(E)** Quantification of P-Ser935 normalized by total LRRK2 amount. Data are presented as mean ± SEM. ^***^P ≤ 0.001 (*n* = 10 independent experiments, one-way ANOVA followed by Bonferroni's multiple comparison test). **(F)** Purified Flag-LRRK2 overexpressed in cells together with Myc-EV or Myc-PAK6 S531N was loaded in SDS-PAGE, transferred onto nitrocellulose membranes and subjected to overlay assay using recombinant 14-3-3γ. Ponceau staining and anti-His antibodies were used to verify LRRK2 loading and bound 14-3-3γ, respectively. **(G)** Quantification of bound 14-3-3γ normalized by total LRRK2 amount. Data are presented as mean ± SEM. ^*^P ≤ 0.05 (*n* = 3 independent experiments, Student's *t*-test analysis).

To translate these findings to a cellular context, we employed BRET to monitor LRRK2/14-3-3γ interaction in the presence or absence of PAK6. To this end, Renilla luciferase-LRRK2 (Rluc-LRRK2) was used as energy donor and YFP-14-3-3γ as energy acceptor (Figure 6A). Coelenterazine was the substrate for the luciferase. Coexpression of Rluc-LRRK2 with YFP-14-3-3γ generated a significant increase in the total BRET signal compared to YFP alone, with YFP emission only occurring when in close proximity (<100 Å) to the luminescent Rluc-LRRK2 (Figure 6B). Instead, coexpression of donor and acceptor proteins with 3xFlag-PAK6 showed a significant reduction of nBRET signal (Figures 6B,C), supporting the notion that PAK6 kinase activity disrupts the LRRK2/14-3-3γ complex. Dissociation of LRRK2/14-3-3 complex in cells results in dephosphorylation of a cluster of serines located at the N-terminal region of LRRK2 (Dzamko et al., 2010; Nichols et al., 2010). To test whether PAK6-mediated 14-3-3γ dissociation from LRRK2 promotes LRRK2 dephosphorylation at these serine residues, we co-expressed LRRK2 wild-type together with PAK6 wild-type, K436M or S531N in HEK293T cells. As predicted, PAK6 induces LRRK2 dephosphorylation at Ser935 in a kinase dependent manner (Figures 6D,E), with PAK6 wild-type inducing 40% reduction of Ser935 phosphorylation relative to empty vector and S531N abolishing Ser935 phosphorylation (Figure 6E). We then tested whether dephosphorylated LRRK2 by PAK6 loses affinity for 14-3-3. LRRK2 purified from cells expressing PAK6 S531N or empty vector was subjected to overlay assays with 14-3-3γ. We observed that PAK6 promotes a complete dephosphorylation of LRRK2 at Ser935, which is no longer competent to bind 14-3-3γ (Figures 6F,G). Taken together, our results demonstrate that PAK6 kinase activity is critical for the regulation of LRRK2/14-3-3γ complex in cells by regulating the constitutive phosphorylation of LRRK2 at Ser935.

### PAK6 rescues LRRK2 G2019S neurite shortening via phosphorylation of 14-3-3γ at Ser59

**Figure 7:**
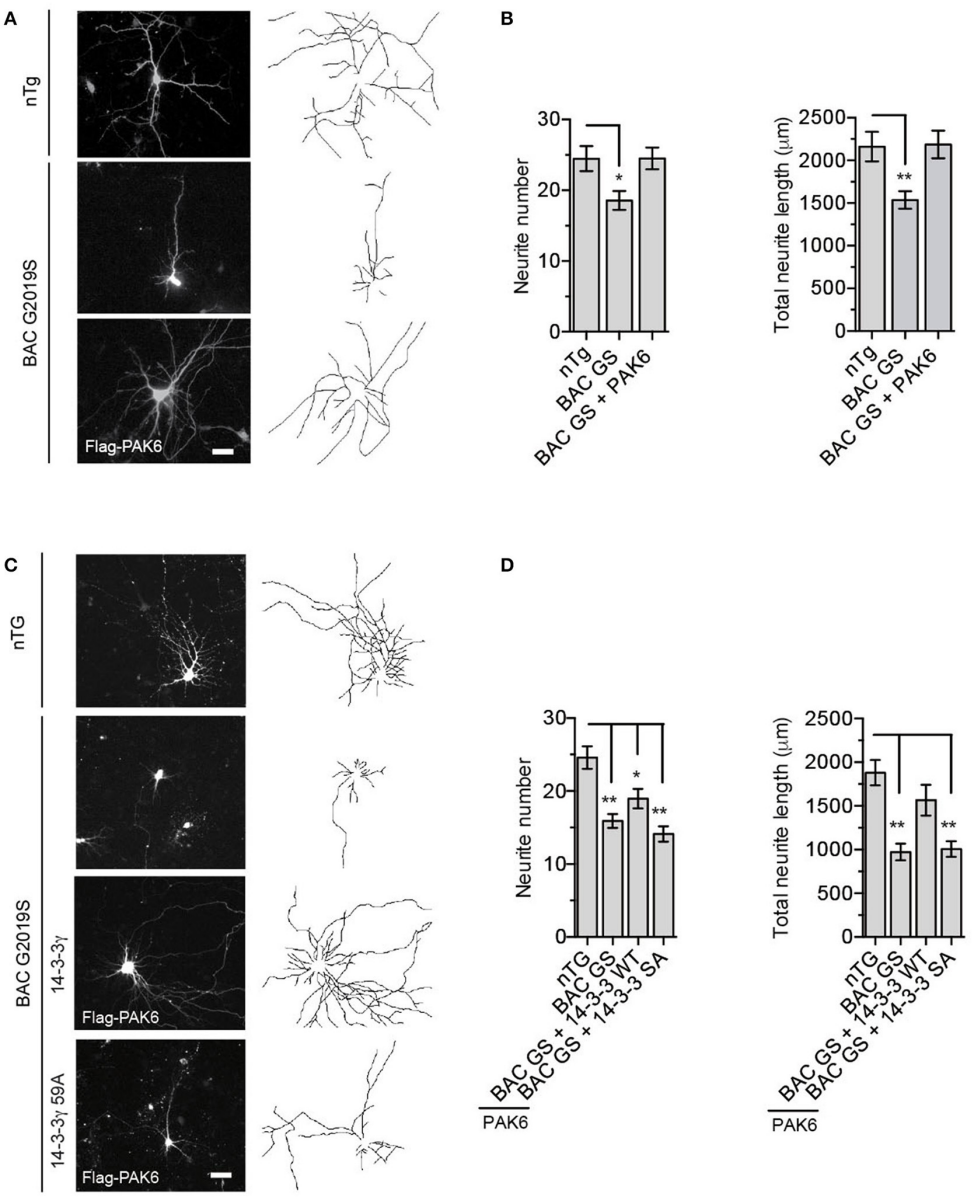
G2019S LRRK2 neurite shortening is restored by PAK6-mediated 14-3-3γ phosphorylation. **(A)** Representative images of primary neurons from nTg and BAC G2019S LRRK2 mice transfected with an empty vector or Flag-PAK6 S531N. GFP was also overexpressed to analyze neuronal branching. **(B)** Quantification of neurite length and number using NeuronJ (ImageJ plugin). Data are presented as mean ± SEM. ^*^P ≤ 0.05 and ^**^P ≤ 0.01 (*n* = 3 independent primary cultures, *n* = 50–70 neurons traced, two-way ANOVA followed by Tukey's multiple comparison test). **(C)** Representative images of primary neurons from nTg and BAC G2019S LRRK2 mice transfected with an empty vector or Flag-PAK6 S531N together with YFP-114-3-3γ wild-type and S59A. mCherry was also overexpressed to analyze neuronal branching. **(D)** Quantification of neurite length and number using NeuronJ (ImageJ plugin). Data are presented as mean ± SEM. ^*^P ≤ 0.05 and ^**^P ≤ 0.01 (*n* = 3 independent primary cultures, *n* = 30–40 neurons traced, two-way ANOVA followed by Tukey's multiple comparison test).

To explore the functional implication of these findings in a neuronal context, we asked whether PAK6 phosphorylation of 14-3-3γ contributes to the previously established link between LRRK2 and PAK6 in regulating neurite outgrowth (Civiero et al., 2015). First, we confirmed that PAK6 phosphorylates 14-3-3γ at Ser59 in primary neuronal cells. To this end, we co-transfected YFP-14-3-3γ together with PAK6 S531N in primary neurons at DIV3 and applied immunocytochemistry analysis at DIV7. We observed that PAK6 clearly enhances the tone of phosphorylation of 14-3-3γ at Ser59 in neurons [Supplemental File (Datasheet 1): Figure S5]. We previously established that PAK6 promotes neurite complexity in a kinase-dependent manner and LRRK2 is required for its function (Civiero et al., 2015). Since the PD-linked LRRK2-G2019S mutant is associated with shorter neurite development in culture (MacLeod et al., 2006; Winner et al., 2011; Sepulveda et al., 2013), we then tested whether active PAK6 rescues the G2019S-associated neurite shortening phenotype in BAC G2019S mice overexpressing murine LRRK2-G2019S. We prepared primary cortical neurons from both non-transgenic (nTg) and BAC-LRRK2-G2019S mice and quantified neuronal branching at DIV7 as previously described (Civiero et al., 2015). Similar to previous results, LRRK2-G2019S neurons display a significant decrease in neurite number and length compared to nTg and, interestingly, ectopic expression of PAK6 S531N almost completely rescued the neurite shortening phenotype (Figures 7A,B). To investigate whether the ability of PAK6 to recover the neurite outgrowth defects of G2019S neurons was dependent on the presence of “phosphorylatable” 14-3-3γ, we expressed ectopic YFP-14-3-3γ wild-type and YFP-14-3-3γ S59A in the presence of PAK6 in BAC G2019S neurons. We observed that PAK6-mediated rescue occurs only in the presence of wild-type 14-3-3γ but not when phospho-deficient 14-3-3γ was present (Figures 7C,D). In summary, these experiments provide evidence that PAK6 phosphorylates 14-3-3γ in neurons at Ser59 and rescues the PD-linked G2019S LRRK2 neurite shortening phenotype through phosphorylation of 14-3-3γ at Ser59.

## Discussion

P21-activated kinases comprise a family of serine-threonine kinases involved in multiple signal transduction pathways, the majority of them affecting cytoskeletal dynamics (Civiero and Greggio, 2017; Kumar et al., 2017). They are divided in two groups according to their activation mechanism, either dependent (group I, PAK1-3) or independent (group II, PAK4-6) of small GTPase binding (Kumar et al., 2017). Group II PAKs are emerging as important players in brain physiology and pathology, particularly in PD. A constitutively active form of PAK4 was found to protect the dopaminergic neurons in 6-hydroxydopamine and α-synuclein rat models of PD (Longo et al., 2017). In addition, our recent work indicates that the PD-associated kinase LRRK2 interacts with PAK6 to promote neurite outgrowth and PD brains show increased PAK6 phosphorylation (Civiero et al., 2015). In the attempt of identifying novel PAK6 effectors relevant for brain pathophysiology, we undertook an unbiased proteomic approach and found that 14-3-3s are high-affinity PAK6 interactors. 14-3-3s were previously suggested to interact with group II PAKs (Tinti et al., 2014), but the functional significance of this interaction has not been investigated. Here, we provide substantial evidence that PAK6 engages in a high-affinity interaction with 14-3-3γ through its autophosphorylation sites Ser113 and Ser560. The clear link between PAK6 kinase activity and its affinity for 14-3-3s prompted us to test PAK6 as a direct kinase for 14-3-3s. Using independent techniques, we found that PAK6 phosphorylates 14-3-3γ within group II PAK consensus sites (Ser59 and Thr145) *in vitro* and in cells. Phosphorylation seems to be the most important way of regulating 14-3-3s activity, affecting their interaction with partners or inducing dissociation of 14-3-3 dimers (reviewed in Aitken, 2011). We showed that PAK6-mediated phosphorylation at Ser59, but not at Thr145, decreases the affinity of 14-3-3γ for client proteins in the brain. Similarly, it has been previously demonstrated that phosphorylation of 14-3-3ζ by PKA at Ser58 (equivalent to Ser59 in 14-3-3γ) interferes with p53 interaction (Gu et al., 2006). Ser58(59) is located at the dimer interface and phosphorylation at this site was proposed to interfere with dimer formation with consequent impact on the binding with client proteins (Woodcock et al., 2003; Gu et al., 2006). The same mechanism was reported for phosphorylated 14-3-3ω at the equivalent residue in plants (Denison et al., 2014). In line with these studies, our results show a shift of 14-3-3γ toward a size compatible with that of a monomer in blue native gels upon PAK6 phosphorylation *in vitro* or by mimicking phosphorylation with S59D but not with S145D. By inspecting the 3D structure of 14-3-3γ, the residue Ser58(9) is positioned in the inner interface of the dimer and therefore it almost inaccessible for modification, whilst Thr145 is positioned on the protein surface. As proposed by others, we cannot exclude that a hierarchical phosphorylation is required (e.g., a first phosphorylation at Thr145 might affect the monomer/dimer equilibrium allowing a second phosphorylation at Ser59). Interestingly, dimer-incapable 14-3-3 proteins retained binding capacity and specificity toward some phospho-partners, and also demonstrated increased chaperone-like activity on various substrates (reviewed in Sluchanko and Gusev, 2012). Therefore, the general decreased affinity of phospho-14-3-3γ and S59D 14-3-3γ for partners could be due to the insertion of a negative charge at Ser59. Indeed, Ser58 is located in close proximity of residues directly involved with phospho-ligand binding pocket.

As a proof of concept of our findings, we demonstrated that PAK6-mediated 14-3-3γ phosphorylation at Ser59 decreases its affinity *in vitro* and in cells for its well-known partner LRRK2. PAK6-induced loss of 14-3-3 binding on LRRK2 results in LRRK2 dephosphorylation at Ser935, one of the LRRK2 phosphosite protected by the chaperone. Compelling evidence revealed that the interaction of LRRK2 with 14-3-3 proteins prevents dephosphorylation of Ser910/Ser935 and stabilizes LRRK2 structure, possibly by influencing LRRK2 dimerization (reviewed in Civiero and Greggio, 2017). Moreover, the ability to interact with 14-3-3 correlates with the pattern of intracellular LRRK2 distribution (Dzamko et al., 2010). However, upstream modulators of the LRRK2/14-3-3 complex and the functional significance of the LRRK2 release have not been identified yet. In addition to phospho-Ser910/935/955/973 clustered at the N-terminal of LRR domain, 14-3-3s bind LRRK2 at phospho-Ser1444 within the ROC-GTPase domain, with 14-3-3γ isoform displaying the highest affinity (Muda et al., 2014). The ROC domain is autophosphorylated by the kinase domain of LRRK2 and this modification is believed to regulate its GTPase activity (Greggio et al., 2009; Gloeckner et al., 2010; Liu et al., 2016). According to this model, ROC may represent the signaling output of LRRK2 and modulation of 14-3-3γ binding may be implicated in controlling LRRK2 cellular cascades. One of best characterized phenotype caused by mutant LRRK2 in neurons is a reduction of neurite complexity (MacLeod et al., 2006; Sepulveda et al., 2013; Matikainen-Ankney et al., 2016), which may represent an early event before neuronal degeneration and motor dysfunction observed in some PD-LRRK2 models (Li et al., 2010; Melrose et al., 2010; Winner et al., 2011; Longo et al., 2014; Beccano-Kelly et al., 2015; Yue et al., 2015; Adeosun et al., 2017). Here we used the BAC-mLRRK2G2019S mouse (Li et al., 2010), which was previously shown to display the neurite shortening phenotype (Sepulveda et al., 2013) and to show signs of neurodegeneration in the *substantia nigra pars compacta* at 18 months of age (Chen et al., 2017), to investigate the potential implication of our findings in LRRK2-PD. We found PAK6 as an upstream modulator of LRRK2/14-3-3 complex promotes neurite outgrowth by its activity and rescues the G2019S LRRK2-induced neurite shortening phenotype through Ser59 phosphorylation of 14-3-3γ. It is well-established that (1) G2019S LRRK2 displays higher kinase activity *in vitro* and *in vivo* (Greggio et al., 2006; Sheng et al., 2012) and (2) alanine substitutions at Ser910/935 interfere with 14-3-3 binding and decrease LRRK2 activity toward substrates *in vitro* (Ito et al., 2016). Putting these two observations together, it is possible that active PAK6 normalizes LRRK2-G2019S activity by modulating LRRK2-14-3-3 binding. Based on our findings, PAK6 kinase activity results in LRRK2 Ser935 dephosphorylation similar to the effect of direct LRRK2 kinase inhibition, providing protection against G2019S-associated phenotypes. As safety liabilities of LRRK2 kinase inhibition have been recently reported (Fuji et al., 2015), identifying alternative targets—such as PAK6—that may provide a safer approach than direct LRRK2 inhibition, is of great value for future therapeutic developments.

Both LRRK2 and PAK6 are kinases upstream the regulation of actin-cytoskeleton dynamics and we previously found that they cooperate to promote neurite complexity in mammalian brain via the LIMK1 pathway (Civiero et al., 2015). It has been proposed that the expression of 14-3-3θ regulates mutant LRRK2 activity and promotes neurite outgrowth (Lavalley et al., 2016). In addition, Kaplan et al. reported that 14-3-3ζ intervenes in axon regeneration and induces neurite branching by interacting with client proteins (Kaplan et al., 2017). Both studies conclude that the beneficial effect of 14-3-3θ and 14-3-3ζ on neurons is disrupted by interfering with general binding of 14-3-3s. These data partially contrast our finding regarding the ability of PAK6 to release 14-3-3γ from LRRK2 and rescue LRRK2-linked pathogenic phenotype, suggesting that a fine balance of 14-3-3-bound and 14-3-3-free targets is probably required to maintain cellular homeostasis and that different isoforms cover specific functions in the cell.

Collectively, our study identifies a novel PAK6-mediated pathway in neurons that involves the phosphorylation of Ser59 in 14-3-3γ and the consequent regulation of LRRK2-14-3-3γ complex. We have previously published that brains from G2019S LRRK2 PD patients display increased PAK6 activation (Civiero et al., 2015). In light of the present findings, we can speculate that PAK6 is hyperactivated in PD brains as a feedback response to normalize LRRK2 activity via 14-3-3γ phosphorylation. Another possibility that deserves future investigations is that LRRK2 kinase activity directly or indirectly promotes PAK6 autophosphorylation. Of note, an increased trend of phospho-Ser58 in 14-3-3s was observed in LRRK2 G2019S mouse brains (Lavalley et al., 2016). However, additional studies are necessary to establish whether Ser59 phosphorylation of 14-3-3γ (and possibly of other isoforms) can be envisaged as a valuable marker and/or therapeutic target in LRRK2 and sporadic PD.

## Author contributions

LC: Designed and performed the majority of the experiments; SC: performed the interaction experiments; AK: Performed the overlay assays and neurite analysis; CM: Performed the BRET experiments and analysis; IT: Prepared the reagents; EL and VB: Helped with the phosphorylation experiments and data analysis; J-MT and M-CC-H: Contributed with data interpretation; CF: Performed the LC-MS/MS analysis and GA interpreted the results; GP: Helped with the phosphorylation experiments and contributed with data interpretation; PL and MC: Contributed with data interpretation and analysis; LB: Contributed with experimental design, data analysis and interpretation; PP: Designed and analyzed the BRET experiments; LC and EG: Conceived the study, designed the experiments and analyzed the data. LC and EG: Wrote the paper with contribution from all authors. All authors read and approved the final manuscript.

### Conflict of interest statement

The authors declare that the research was conducted in the absence of any commercial or financial relationships that could be construed as a potential conflict of interest.

## Abbreviations

LRRK2: Leucine-rich repeat kinase 2
PD: Parkinson's disease
PAK6: P21 (RAC1) Activated Kinase 6
CRIB: Cdc42 and Rac-interactive binding
BCR: breakpoint cluster region protein
PKA: protein kinase A
CK1α: casein kinase 1 alpha
IKKs: IκB kinases
PP1: protein phosphatase 1
BRET: bioluminescence resonance energy transfer
DIV: days *in vitro*
EDTA: ethylenediaminetetraacetic acid
TEMED: *N,N,N*′,*N*′ tetramethylethylenediamine
APS: ammonium persulfate
PVDF: polyvinylidenedifluoride
CAN: acetonitrile
DTT: dithiothreitol
TBS-T: Tris-buffered saline plus 0.1% Triton
BSA: bovine serum albumin
PFA: paraformaldehyde
SEM: standard error of the mean
LC-MS/MS: liquid chromatography/tandem mass spectrometry
DMSO: dimethyl sulfoxide
nTg: non-transgenic.
