# Supplementary material for: PAK6 Phosphorylates 14-3-3γ to Regulate Steady State Phosphorylation of LRRK2
Source: Front Mol Neurosci. 2017 Dec 14;10:417. doi: 10.3389/fnmol.2017.00417 (PMC5735978; doi:10.3389/fnmol.2017.00417)
Supplement: Supplementary file 1 [file DataSheet1.pdf]

Supplementary Material

PAK6 phosphorylates 14-3-3γ to regulate steady state phosphorylation of LRRK2

Laura Civiero, Susanna Cogo, Anneleen Kiekens, Claudia Morganti, Isabella Tessari, Evy Lobbestael, Veerle Baekelandt, Jean-Marc Taymans, Marie-Christine Chartier-Harlin, Cinzia Franchin, Giorgio Arrigoni, Patrick A. Lewis, Giovanni Piccoli, Luigi Bubacco, Mark R. Cookson, Paolo Pinton and Elisa Greggio

*** Correspondence:** laura.civiero@unipd.it or elisa.greggio@unipd.it. Department of Biology, University of Padova, Via Ugo Bassi 58/B, 35131, Padova, Italy

Figure S1. Ser113 and Ser560 are autophosphorylation sites in PAK6.

(A) *In vitro* kinase assays of Flag-PAK6 wild-type, S113A and S560A in the presence of the general Ser/Thr substrate myelin basal protein (MPB, 10 µg) in the presence of ^{33}P-ATP/Mg^{2+} for 1 hour. Samples were subjected to SDS-PAGE and transferred onto nitrocellulose membranes. The upper indicates PAK6 autophosphorylation and MBP phosphorylation (phosphoscreen). Total protein amount was verified coomassie blue staining of the membrane (lower panels).

(B) Quantification of incorporated ^{33}P-radioactivity normalized by total protein (n=4 experiments). Data are presented as mean ± SEM. Two-way ANOVA followed by Tukey's multiple comparison test (*P≤0.05, **P≤0.01, ***P≤0.001, and ****P≤0.0001).

Figure S2. LC-MS/MS analysis reveals that PAK6 phosphorylates 14-3-3γ at multiple sites.

(A) Annotated MS/MS spectrum relative to the doubly charged ion at m/z=593.27997 Da corresponding to the phosphopeptide 143RATVVESSEK152. Mass difference between theoretical and experimental m/z is 0.52 ppm. Theoretical fragment ions matching the sequence are marked in red (for b and a series) and in blue (for y series).

(B) Annotated MS/MS spectrum relative to triply charged ion at m/z=751.39447 Da corresponding to the phosphopeptide 51NVVGARRSSWRVISSIEQK69. Mass difference between theoretical and experimental m/z is 0.56 ppm. Theoretical fragment ions matching the sequence are marked in red (for b and a series) and in blue (for y series).

Figure S3. PAK6 phosphorylates S59 not S58 of 14-3-3γ.

In vitro non-radioactive kinase assays of recombinant 14-3-3γ wild-type, S58A, S59A and S58A/S59A in the presence of immunopurified PAK6 wild-type, showing that S59, not S58, is phosphorylated by PAK6. Upper panel represent an immunoblot against pan-phosphoSer58/59 14-3-3; lower panel indicates total protein loading (Ponceau staining).

Figure S4. LRRK2 interacts with a subset of 14-3-3s including 14-3-3γ.

(A) Recombinant 14-3-3 isoforms expressed and purified from E. coli (coomassie staining).

(B) Immunopurified Flag-LRRK2 and Flag-PAK6 were loaded into SDS-PAGE at equimolar concentration, transferred onto nitrocellulose membranes and subjected to overlay assays using all recombinant 14-3-3 isoforms. Anti-Flag and anti-His antibodies were used to verify LRRK2 and PAK6 loading (right panel) and bound 14-3-3, respectively. SDS-PAGE/transfers were done in the same gel/membrane and membranes were subsequently cut to allow incubations with the different 14-3-3 isoforms. Western blotting and detection was performed in parallel to allow quantitative comparison.

© Quantification of bound 14-3-3 normalized by total LRRK2 and PAK6 amount (n=3 independent experiments).

Figure S5. Active PAK6 phosphorylates 14-3-3γ in neurons.

Primary cortical neurons were cotransfected with PAK6 S531N or empty vector and 14-3-3γ at DIV4. Cells were fixed at DIV7 and stained with anti-14-3-3 (green), pSer58/59 pan antibody (red) and PAK6 (blue).

Figure S1

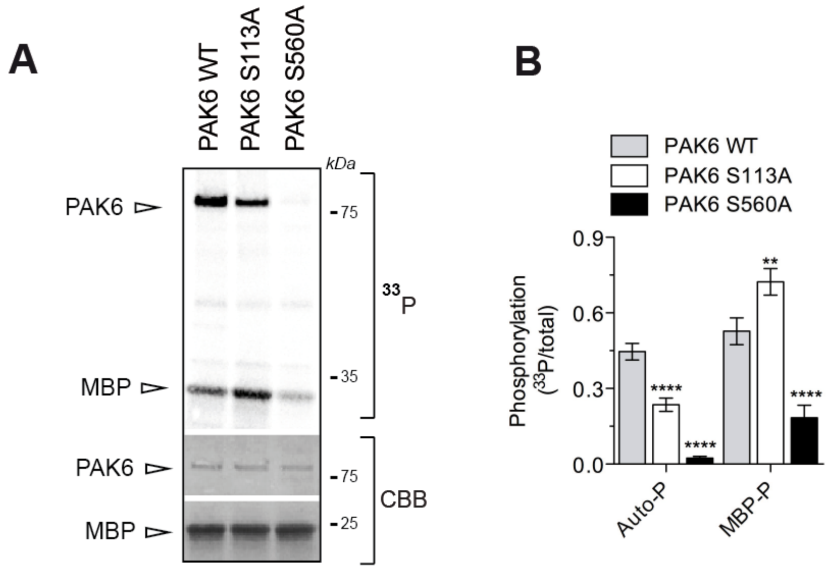
A
PAK6 WT
PAK6 S113A
PAK6 S560A
kDa
PAK6
75
33P
MBP
35
PAK6
75
CBB
MBP
25
B
PAK6 WT
PAK6 S113A
PAK6 S560A
Phosphorylation (33P/total)
0.9
0.6
0.3
0.0
**
****
****
****
Auto-P
MBP-P

Figure S2

A

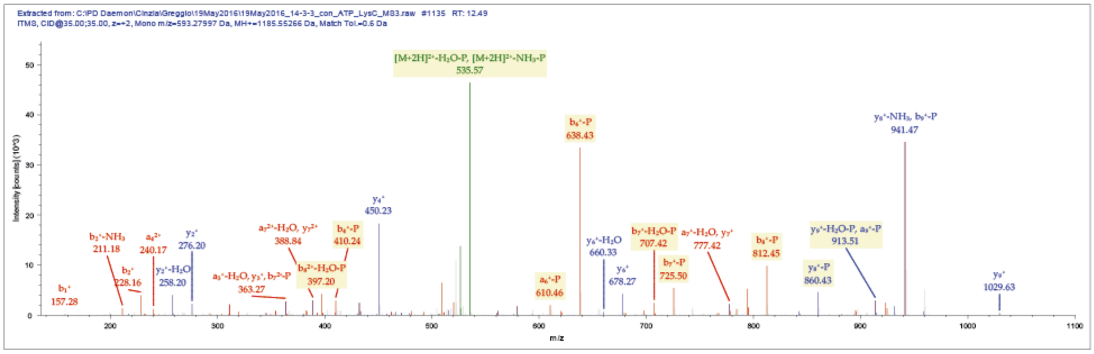

#1	a⁺	a²⁺	b⁺	b²⁺	Seq.	y⁺	y²⁺
1	129.11348	65.06038	157.10840	79.05784	R		
2	200.15060	100.57894	228.14552	114.57640	A	1029.45004	515.22866
3	381.16461	191.08594	409.15953	205.08340	T-Phospho	958.41292	479.71010
4	480.23303	240.62015	508.22795	254.61761	V	777.39891	389.20309
5	579.30145	290.15436	607.29637	304.15182	V	678.33049	339.66888
6	708.34405	354.67566	736.33897	368.67312	E	579.26207	290.13467
7	795.37608	398.19168	823.37100	412.18914	S	450.21947	225.61337
8	882.40811	441.70769	910.40303	455.70515	S	363.18744	182.09736
9	1011.45071	506.22899	1039.44563	520.22645	E	276.15541	138.58134
10					K	147.11281	74.06004

B

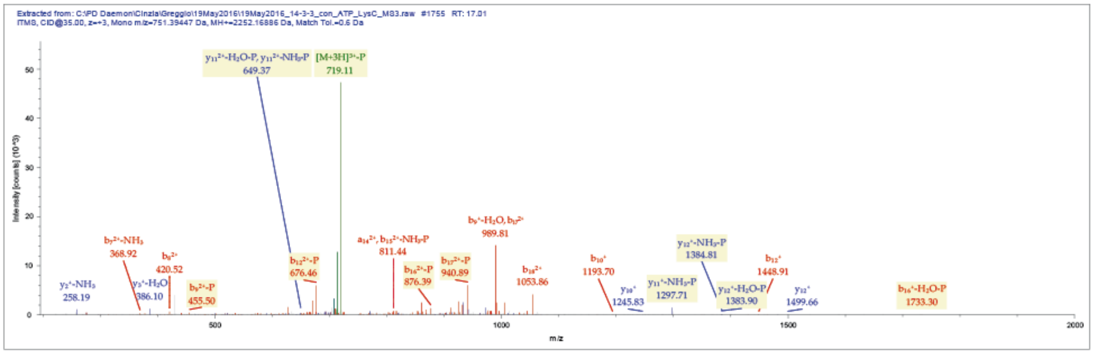

#1	a⁺	a²⁺	a³⁺	b⁺	b²⁺	b³⁺	Seq.	y⁺	y²⁺	y³⁺	#2
1	87.05529	44.03128	29.68995	115.05021	58.02874	39.02159	N				19
2	186.12371	93.56549	62.71276	214.11863	107.56295	72.04439	V	2138.12311	1069.56519	713.37922	18
3	285.19213	143.09970	95.73556	313.18705	157.09716	105.06720	V	2039.05469	1020.03098	680.35642	17
4	342.21360	171.61044	114.74272	370.20852	185.60790	124.07436	G	1939.98627	970.49677	647.33361	16
5	413.25072	207.12900	138.42176	441.24564	221.12646	147.75340	A	1882.96480	941.98604	628.32645	15
6	569.35184	285.17956	190.45547	597.34676	299.17702	199.78710	R	1811.92768	906.46748	604.64741	14
7	725.45296	363.23012	242.48917	753.44788	377.22758	251.82081	R	1655.82656	828.41692	552.61371	13
8	812.48499	406.74613	271.49985	840.47991	420.74359	280.83149	S	1499.72544	750.36636	500.58000	12
9	979.48335	490.24531	327.16597	1007.47827	504.24277	336.49761	S-Phospho	1412.69341	706.85034	471.56932	11
10	1165.56267	583.28497	389.19241	1193.55759	597.28243	398.52405	W	1245.69505	623.35116	415.90320	10
11	1321.66379	661.33553	441.22612	1349.65871	675.33299	450.55775	R	1059.61573	530.31150	353.87676	9
12	1420.73221	710.86974	474.24892	1448.72713	724.86720	483.58056	V	903.51461	452.26094	301.84305	8
13	1533.81628	767.41178	511.94361	1561.81120	781.40924	521.27525	I	804.44619	402.72673	268.82025	7
14	1620.84831	810.92779	540.95429	1648.84323	824.92525	550.28593	S	691.36212	346.18470	231.12556	6
15	1707.88034	854.44381	569.96497	1735.87526	868.44127	579.29660	S	604.33009	302.66868	202.11488	5
16	1820.96441	910.98584	607.65966	1848.95933	924.98330	616.99129	I	517.29806	259.15267	173.10420	4
17	1950.00701	975.50714	650.67386	1978.00193	989.50460	660.00549	E	404.21399	202.61063	135.40951	3
18	2078.06559	1039.53643	693.36005	2106.06051	1053.53389	702.69169	Q	275.17139	138.08933	92.39531	2
19							K	147.11281	74.06004	49.70912	1

Figure S3

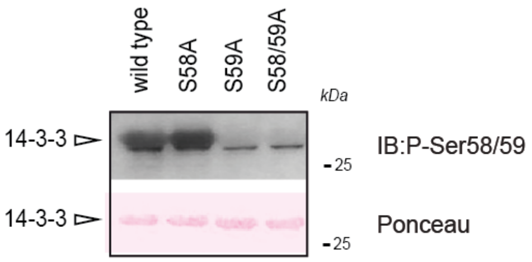
wild type
S58A
S59A
S58/59A
kDa
14-3-3
IB:P-Ser58/59
25
14-3-3
Ponceau
25

Figure S4

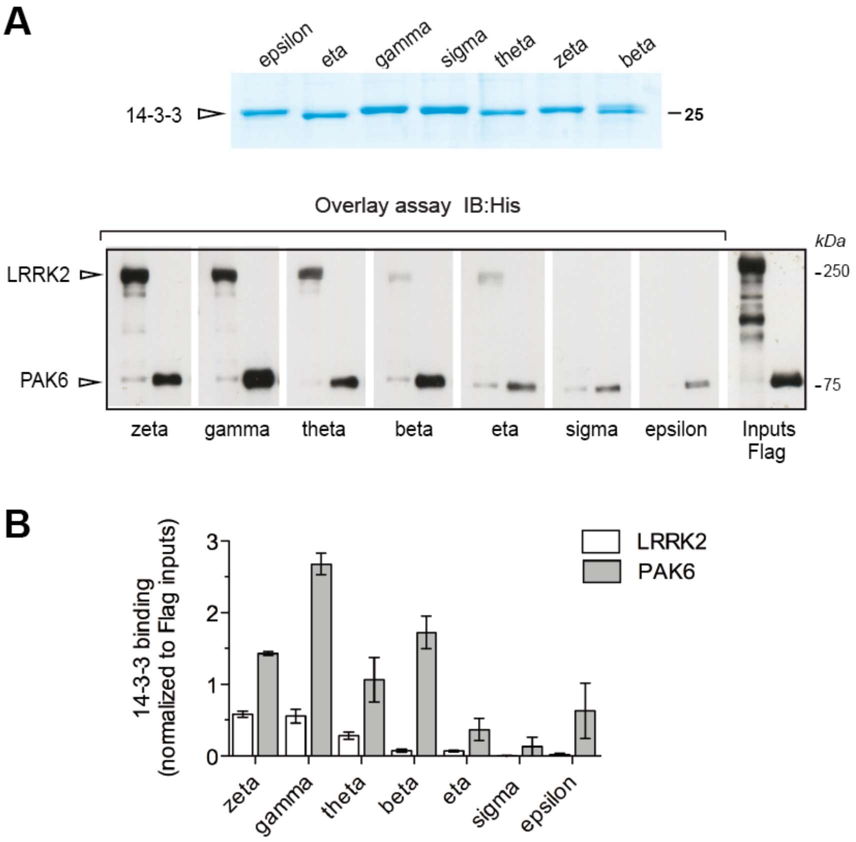
A
epsilon
eta
gamma
sigma
theta
zeta
beta
14-3-3
25
Overlay assay IB:His
kDa
LRRK2
PAK6
250
75
zeta
gamma
theta
beta
eta
sigma
epsilon
Inputs
Flag
B
14-3-3 binding
(normalized to Flag inputs)
3
2
1
0
LRRK2
PAK6
zeta
gamma
theta
beta
eta
sigma
epsilon

Figure S5

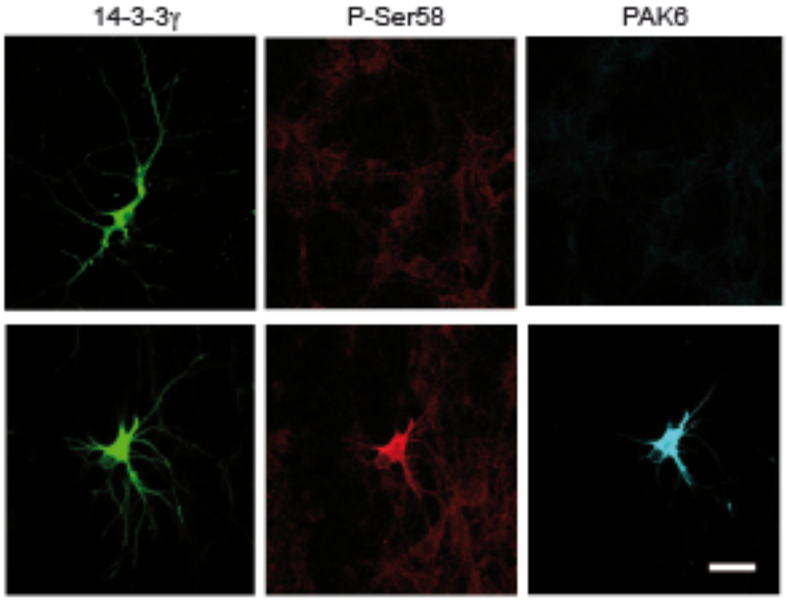

Supplementary Table 1. 14-3-3g phospho-peptides revealed by LC-MS/MS.

Summary of all peptides recovered upon three independent runs of LC-MS/MS of 14-3-3g phosphorylated *in vitro* by PAK6 S531N.

14-3-3γ phospho-peptides
RA**pT**VVESSEK
AY**pS**EAHEISK
NVTELNEPLSNEERNLL**pS**VAYK
**pT**AFDDAIAELDTLNEDSYK - TAFDDAIAELD**pT**LNEDSYK
NVVGARR**pS**SWRVISSIEQK - NVVGARRS**pS**WRVISSIEQK - NVVGARRSSWRVI**pS**SIEQK - NVVGARRSSWRVIS**pS**IEQK
